# Echocardiographic Assessment of Left Ventricular Systolic and Diastolic Functions in Dogs with Severe Sepsis and Septic Shock; Longitudinal Study

**DOI:** 10.3390/ani11072011

**Published:** 2021-07-05

**Authors:** Mehmet Ege Ince, Kursad Turgut, Amir Naseri

**Affiliations:** 1 Department of Internal Medicine, Faculty of Veterinary Medicine, Near East University, 99100 Nicosia, North Cyprus, Turkey; kursad.turgut@neu.edu.tr; 2 Department of Internal Medicine, Faculty of Veterinary Medicine, Selcuk University, 42130 Konya, Turkey; anaseri@selcuk.edu.tr

**Keywords:** systolic dysfunction, diastolic dysfunction, severe sepsis, septic shock, dogs

## Abstract

**Simple Summary:**

Sepsis is associated with cardiovascular changes. The aim of the study was to determine sepsis-induced myocardial dysfunction in dogs with severe sepsis and septic shock using transthoracic echocardiography. Clinical, laboratory and cardiologic examinations for the septic dogs were performed at admission, 6 and 24 h, and on the day of discharge from the hospital. Left ventricular (LV) systolic dysfunction, LV diastolic dysfunction, and both types of the dysfunction were present in 13%, 70%, and 9% of dogs with sepsis, respectively. Dogs with LV diastolic dysfunction had a worse outcome and short-term mortality. Transthoracic echocardiography can be used for monitoring cardiovascular dysfunction in dogs with sepsis.

**Abstract:**

The purpose of this study was to monitor left ventricular systolic dysfunction (LVSD) and diastolic dysfunction (LVDD) using transthoracic echocardiography (TTE) in dogs with severe sepsis and septic shock (SS/SS). A prospective longitudinal study using 23 dogs with SS/SS (experimental group) and 20 healthy dogs (control group) were carried out. All the clinical, laboratory and cardiologic examinations for the experimental dogs were performed at admission, 6 and 24 h after the start of treatment and on the day of discharge. LVSD was described as LV ejection fraction (LVEF) < 50%. LVDD was determined when the septal mitral annulus early diastolic velocity (LVEm) was <8 cm/s. LVSD and LVDD were present in 3 and 16 dogs with SS/SS, respectively, with both types of dysfunction present in 2 of the dogs. Although all the dogs with LVSD survived, 8 dogs with LVDD did not. The survival period was significantly shorter in dogs with an LVEm < 8 cm/s (1.3 ± 1.4 days). In conclusion, LVDD, rather than LVSD, was a common cardiovascular abnormality in the septic dogs, and this may be a negative prognostic factor. TTE is a useful tool for the identifying and monitoring of myocardial dysfunction in the dogs with SS/SS.

## 1. Introduction

Sepsis is a life-threatening syndrome. It happens when the immune system overreacts to an infection and causes organ dysfunctions [1]. Canine parvovirus infection (CPVI) causes sepsis-induced myocardial dysfunction (SiMD) in dogs [2,3,4].

Previous studies in human medicine showed that severe sepsis and septic shock (SS/SS) are characterized with absolute or relative dehydration [5], left ventricular systolic dysfunction (LVSD) and left ventricular diastolic dysfunction (LVDD) [6,7,8] and right ventricular (RV) dysfunction [9], marked peripheral vasodilation [10], and vasoplegia [11]. Transthoracic echocardiography (TTE) has been used for guiding hemodynamic management of cardiac function in human patients, dogs, and calves in the intensive care unit (ICU) [4,12,13,14]. SiMD is a reversible dysfunction causing both LVSD and LVDD in humans [15]. The various forms of SiMD can present either in isolation or as a combination of the various forms. These different forms of SiMD may be reversible, if the appropriate treatments are administered in a timely manner [16]. The studies on SiMD show that LVDD, when compared to LVSD, is associated with a higher mortality rate in humans [8,12].

The importance of LV dysfunction in dogs with critical illness were evaluated [17]. Ince et al. [4] have shown that pulsed wave tissue doppler imaging (PW-TDI) septal mitral annulus systolic velocity (LVSm) and PW-TDI septal mitral annulus early diastolic velocity (LVEm) are useful prognostic indices of LVSD and LVDD in dogs with SS/SS, respectively. In septic bovine calves, low LVEDVI and low LVESVI was the most clinically important circulatory dysfunction [13].

According to our knowledge, there are no studies that have evaluated the use of TTE to assess LVSD and LVDD in dogs with SS/SS, in a longitudinal study. We hypothesized that LVSD and LVDD can develop in dogs with SS/SS and serial echocardiographic examinations (EExs) can be helpful in monitoring cardiovascular dysfunction. Accordingly, the objective of the study was to investigate systolic and diastolic function of the LV by TTE during hospitalization period in dogs with CPVI.

## 2. Materials and Methods

We had ethics committee approval from the Faculty of Veterinary Medicine, the University of Selcuk for this study (permit number: 2015/03). Records of dogs that were admitted to Selcuk University Hospital for Animals were evaluated from December 2016 to December 2019.

### 2.1. Animals

Twenty-three dogs suffering from CPVI with SS/SS (experimental group) and 20 healthy dogs (control group), ≤6 months of age, incorporating both sexes and different breed types, were included.

### 2.2. Control Dogs

The control dogs, which were brought to the clinic for vaccination, were deemed healthy according to clinical and laboratory examinations and a negative commercial SNAP CPV antigen test (IDEXX, SNAPshot Dx, Westbrook, ME, USA). All the clinical, laboratory, and cardiologic examinations for the control dogs were performed once when they were brought to our clinic. The clinical examination findings, complete blood count (CBC) results and serum biochemistry analyses were all within the reference ranges. The 3-min ECG recordings and EEx results of the control dogs were also normal.

### 2.3. Experimental Dogs

The experimental dogs had clinical signs (vomiting and/or bloody diarrhea) compatible with CPVI. All SNAP CPV antigen test were positive, and none of them had been vaccinated with commercial parvovirus vaccine. All the clinical, laboratory and cardiologic examinations for the experimental dogs were performed at admission, plus 6 and 24 h from the start of the treatment and on the day of discharge (D of D). Inclusion criteria in the experimental group were the recognition of systemic inflammatory response syndrome (SIRS) and SS/SS. The dogs with congenital heart diseases, poor echocardiographic images, and measurements, and had received any other therapy (e.g., fluid therapy, vasopressor, positive inotropic agent) were excluded from study.

### 2.4. Criteria for Definition SIRS, Severe Sepsis, and Septic Shock

Sepsis was defined as the existence of SIRS and a positive SNAP CPV antigen test. Definitions for SIRS were based on the presence of the two or more of the following abnormalities: leukopenia (<6000 cells/μL) or leukocytosis (>16,000 cells/μL), abnormal rectal temperature (<37.8 °C or >39.4 °C), tachycardia (>140 beats per minute), and tachypnea (>30 breaths per minute or pCO2 < 32 mmHg) [18].

Severe sepsis was defined as sepsis associated with one or more of the following: organ dysfunction, hypoperfusion, or hypotension. When severe sepsis did not respond to one bolus of IV fluid administration, it was regarded as septic shock and required vasopressor therapy. Hypotension was defined as systolic blood pressure (SBP) <90 mmHg and mean blood pressure (MBP) <70 mmHg [19]. Dehydration rate was determined as mild (<5%), moderate (<8%) and severe (>10%) [20]. The experimental dogs were monitored (SBP and MAP, ECG recordings) in the ICU (Compact 7, Medical Econet GmbH, Oberhausen, Germany).

### 2.5. Laboratory Analyzes

Five mL of blood were collected by vena cephalica veni puncture at the time of admission, 6 h, 24 h, and D of D from the hospital. One mL of the collected sample was anaerobically transferred into sodium heparin containing plastic syringes and blood gas analysis was performed immediately. An extra mL of the blood was put into the tubes containing K3EDTA and CBC analysis was performed immediately. The remaining 3 mL of collected blood was put into the tubes without anticoagulant, centrifuged at 2000× *g* for 5 min at 4 °C. Serum samples were extracted for biochemical analyses and enzyme-linked immunosorbent assay (ELISA) analysis.

Venous blood gas analysis which included pH, the partial pressure of carbon dioxide (pCO2), partial pressure of oxygen (pO2), lactate, sodium (Na), potassium (K), glucose, base excess (BE), and bicarbonate (HCO3) was performed using an automatic blood gas analyser (GEM Premier 3000, Instrumentation Laboratory, Lexington, MA, USA). CBCs including total leukocytes, lymphocytes, granulocytes, monocytes, erythrocytes, mean corpuscular volume (MCV), hematocrit (HCT), mean corpuscular haemoglobin concentration (MCHC), haemoglobin (Hgb), and thrombocyte were done using an automatic cell counter (MS4e, Melet Schlosing Laboratories, Osny, France). Blood urea nitrogen (BUN), creatinine, alanine aminotransferase (ALT), aspartate aminotransferase (AST), alkaline phosphatase (ALP), albumin and total protein (TP) concentrations were measured by a semi-automatic biochemical analyzer (BT 3000 plus, Biotecnica Instruments S.p.A., Roma, Italy). Serum cardiac troponin I (cTnI) levels were measured according to the manufacturer’s protocol using a canine cTnI commercial ELISA kit (cTnI, MyBioSource, San Diego, CA, USA).

### 2.6. Blood Pressure Measurement

SBP and MAP were determined indirectly using an oscillometric technique (Compact 7, Medical Econet GmbH, Oberhausen, Germany). Blood pressure (BP) measurements were performed in a quiet, isolated area after the dogs with SS/SS has had time to adjust to its surroundings. The cuff was 40% of the limb circumference. The dogs with SS/SS were restrained in lateral recumbency. The first reading was discarded, and the next 5 readings were averaged [19].

### 2.7. Echocardiographic Evaluation

Transthoracic echocardiography (TTE) was performed in the ICU with echocardiographic unit and a 4.0 to 7.0 MHz sector probe (SIUI, Apogee 3500, Guangdong, China). Comprehensive 2-dimensional (2D), M-mode and Doppler EExs were applied on all the control and experimental dogs from the right parasternal views (long- and short-axis); and apical views (4-chamber) [21]. All measurements in dogs with sinus rhythm were taken from 3 cardiac cycles and mean values calculated. Heart rate (HR) was determined at the same time with EExs using a base-apex or lead II electrocardiogram [21,22,23]. All echocardiographic measurements were made by 2 investigators (ICU staff) and were reviewed by one investigator (non-certified cardiologists) reviewing videotape recorded examinations.

#### 2.7.1. M-Mode Echocardiography

M-mode EExs of the LV was performed using a right parasternal long-axis view (5- chamber) and right parasternal short-axis view according to the quality of the window and of the images as described [21,24,25]. In each dog, left ventricular end-diastolic and end-systolic dimensions were measured by M-mode image(s) using a leading edge-to-leading edge technique. The left ventricular internal dimensions were measured at the level of the papillary muscles just below the origin of the chordae tendinae. End diastolic measurements corresponded to the largest diastolic dimension (at the onset of R-wave) and end-systolic measurements corresponded to the smallest systolic dimension (during the T-wave). The investigators obtained measurements from three representative images and were averaged for analyzes [25]. The ECG was used along all measurements [26]. Left ventricular end-diastolic volume (LVEDV), left ventricular end-systolic volume (LVESV), and left ventricular ejection fraction (LVEF) were measured using the Teichholz method using the software program of the echocardiogram as described by Boon [24]. Stroke volume (SV) was calculated as the difference between LVEDV and LVESV. Left ventricular cardiac output (LVCO) determined with heart rate (HR) multiplied by the stroke volume (SV) [26,27]. The LVEDV, LVESV, and LVCO values were indexed according to body surface area, to obtain the LVEDVI, LVESVI, and LVCI [28]. The E-point septal separation (EPSS) were measured using the mitral valve M-mode examination [24].

#### 2.7.2. Doppler Echocardiography

Mitral inflow PW-Doppler measurement of peak E and A waves, and E/A ratio were determined. PW-TDI septal mitral annulus early diastolic (Em) velocity and PW-TDI septal mitral annulus peak systolic (Sm) was obtained [23,24].

#### 2.7.3. Criteria for Systolic and Diastolic Dysfunction

LVSD was described as LVEF <50% [23,27,28]. LV diastolic function was evaluated based on the American Society of Echocardiography (ASE) guidelines [29] and classified as; if Em ≥ 8 cm/s: normal; if Em < 8 cm/s, DT > 200 ms and E/A < 0.8: impaired relaxation; if Em < 8 cm/s, DT 160-200 ms, E/A 0.8-1.5, and E/Em 9-12: pseudonormal; if Em < 8 cm/s, DT < 160 ms, E/A > 2, and E/Em ≥ 13: restrictive.

### 2.8. Electrocardiography (ECG)

A standard six-lead electrocardiogram (VE-300, Edan, Shenzhen China) was performed in right lateral recumbency, and the electrocardiography (ECG) traces were recorded (paper speed: 50 mm second (mm/s); calibration at 1 millivolt (mV) = 1 cm (cm) [30].

### 2.9. Pulse Oximetry

Tissue oxygenation was measured by placing the clamp probe of a pulse oximeter (SpO2) (Compact 7, Medical Econet GmbH, Oberhausen, Germany) to an unpigmented portion of the buccal mucosa of the dog [31].

### 2.10. Treatment Protocol

After taking blood samples and measurements, we employed a standardized treatment protocol that involved fluid therapy, vasoactive medication, antimicrobial therapy, blood products, anticoagulants, venous thromboembolism prophylaxis, stress ulcer prophylaxis, and nutrition. Dogs with SS/SS were monitored to guide the shock treatment using lactate, glucose, SBP and MAP, SpO2, acid-base status, and ECG recordings according to the current understanding of optimal treatment protocols for septic shock [32,33].

Intravenous fluid administration was initiated using 0.9% NaCl solution at 60 mL/kg for the first hour of treatment. Then, multiple (up to four) boluses of 10–20 mL/kg were administered over 10–15 min and the effect on clinical signs (e.g., heart rate, respiratory rate, mucous membrane color, and pulse quality) were monitored. Following this, fluid maintenance therapy was administered using 0.9% NaCl at 20 mL/kg/day. The restoration of intravascular volume, correction of hypoglycemia, was closely monitored. Dextrose (5%) was added to the IV fluids if hypoglycemia was present. In cases with diastolic dysfunction, a colloid solution (hydroxyethyl starch 6%, 10 mL/kg/h, IV) was given to prevent the development positive fluid balance due to the use of large amounts of crystalloid solution.

Broad-spectrum antibiotics and anti-inflammatory treatment were instituted using cefazolin (Sefazol^®^, Mustafa Nevzat, Turkey, 30 mg/kg, IV, every 8 h), enrofloxacin (Dufafloxacin^®^, Holland, 5 mg/kg, IM, every 12), metronidazole (Polgyl^®^, Polifarma, Turkey, 10 mg/kg, IV, every 12 h) and meloxicam (Metacam^®^, Boehringer Ingelheim, Istanbul, Turkey, 0.1 mg/kg, IV, 24 h), respectively. Oxygen (100 mL/kg/min) was applied via a nasal oxygen mask to dogs with SpO2 < 90%. Potassium was supplemented if potassium was less than 3.5 mEq/L [34]. Dalteparin (FRAGMİN^®^, Pfizer, Belgium) was administered at a dose of 100 IU/kg every 8 to 12 h for venous thromboembolism prophylaxis. Fresh whole blood was administered at a dose of 20 mL/kg in dogs with a hematocrit of less than 20% [35]. Vasopressor therapy was administered to the six hypotensive dogs (SBP < 90 mm Hg or MAP < 70 mmHg), despite one bolus of fluid therapy, by infusing norepinephrine (1.5 µg/kg/min in 0.9% NaCl solution without a loading dose. If an adequate clinical response was not achieved after 2 h, the rate of norepinephrine infusion was doubled [4,36].

### 2.11. Statistical Analysis

Data analysis was performed using statistical software (SPSS 25.00 for windows). For determining whether the variables have normal distribution Shapiro-Wilk test was used. Parametric data were evaluated by one-way ANOVA and the post hoc Tukey test as mean ± standard deviation (SD) and non-parametric data were evaluated by man Whitney U test as median (min/max). Categorical variables were evaluated by Chi-square test. Survival was evaluated using a Kaplan–Meier analysis and log-rank tests. Statistical significance was considered as *p* < 0.05.

## 3. Results

### 3.1. Animals

There were no statistical differences between the control dogs and experimental dogs for body weight, ages, and gender. The weights, gender and ages in control dogs were 8.67 ± 5.51 kg, 9 males and 11 females and 3.11 ± 1.07 months, respectively. The weights, gender and ages in experimental dogs were 7.63 ± 4.16 kg, 10 males and 13 females and 3.4 ± 0.7 months, respectively.

### 3.2. Clinical Examinations

Twenty-three dogs with SS/SS hospitalized over 7 days were qualified for our study and underwent serial echocardiographic evaluation. All the dogs with SS/SS fulfilled the criteria for sepsis at the time of admission to the hospital. Severe sepsis and septic shock were determined in 21 (91%) and 2 (9%) dogs with CPVI, respectively. The most common clinical signs in dogs with SS/SS were mental depression, bloody diarrhea, hyperemic mucous membranes, vomiting, moderate or severe dehydration, hypokinetic peripheral pulse quality (PPQ) (Table 1), tachypnoea, tachycardia, prolonged capillary refill time (CRT) (Table 1), and hyperthermia/hypothermia at admission. Dehydration rate was moderate (61% of cases) and severe (39% of cases) at admission. All these clinical parameters had returned to normal by the D of D from the ICU in survived dogs.

The mean HR was significantly elevated (*p* < 0.05) in dogs with SS/SS at admission, and the 6th hour, when compared with the values in the control group, and returned to normal before the D of D (Table 1). There was no difference in SBP and MAP during the study (*p* > 0.05) (Table 1). Hypotension was evident in 10 of 23 dogs with SS/SS at admission. Two of the 23 dogs still had low BP at the 6th hour of the treatment and considered as septic shock. Buccal mucosa SpO2 in septic dogs was lower (at admission, 6th, 24th hours, and on the D of D) than that of healthy dogs during the hospitalization period (*p* < 0.05) (Table 1).

### 3.3. Hematological Examinations

White blood cell (WBC) (decreased at the 6th hour), granulocyte (were low on admission and at the 6th hour), monocyte (were low on admission, and at both 6th and 24th hours) and red blood cell (RBC) (was elevated on admission) counts, HCT (were elevated on admission) and MCV (was elevated on admission) values in dogs with SS/SS were significantly different compared to the control group (*p* < 0.05) (Table 2).

### 3.4. Acid-Base Balance, Biochemical Analysis and cTn I

Venous blood pH in dogs with SS/SS were not significantly different compared the control group (*p* > 0.05) (Table 3). Metabolic acidosis (11/23, 49%) was remarkable with a markedly decreased BE value (at admission and 6th hours) (*p* < 0.05) (Table 3). Lactate concentration in dogs with SS/SS were not significantly different when compared with the value in the control group (*p* > 0.05). High lactate values (>2 mmol/L) were present in 49% (11/23) of cases (Table 3). Serum creatinine and BUN concentrations remained unchanged during the treatment (*p* > 0.05) (Table 3); however, 26% (6/23) dogs had azotemia at the time of admission (BUN > 40 mg/dL creatinine >1.6 mg/dL). Hypoglycemia was detected in %30 (7/23) of dogs with sepsis on admission, but mean glucose concentrations did not change during the study and ranging from 68 to 132 mg/dL (*p* > 0.05) (Table 3). Serum ALT activities were elevated in septic dogs (30%, 7/23) when compared with the control group on admission. Potassium concentration in dogs with SS/SS was significantly lower in comparison with the control group at both 6 and 24 hours (*p* < 0.05) (Table 3). Cardiac troponin I was not significantly different in the experimental group of dogs when compared with the control dogs (*p* > 0.05). The highest value in control dogs was <30 pg/mL. Therefore, it was high in 1 dog with LVSD and 4 dogs with LVDD (>30 pg/mL) at admission, at 6 and 24 hours, and on the D of D (Table 4).

### 3.5. ECG

ECG analysis of dogs with SS/SS revealed sinus tachycardia in a total of 16 dogs (70%) and sinus arrhythmia in 3 cases at admission. When we evaluated the dogs with SS/SS, there were changes in the *p*–QRS–T morphology, 2 dogs had S–T elevation and 2 dogs had tall T waves. Six of the non-survival dogs had sinus tachycardia (2 cases), S–T elevation (2 cases), and tall T wave (2 cases).

### 3.6. Echocardiography

**Figure 1:**
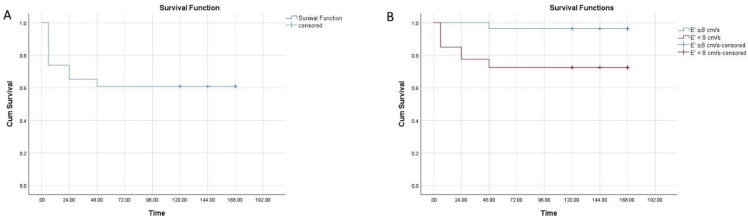
The average time of a survivor was 108 ± 15 h. The cumulative survival probability was 73% ± 9%, 65% ± 9% and 60% ± 10% at the 6th, 24th and 48th hours of the study, respectively (**A**), and the survival period was significantly shorter in patients with an PW-TDI septal mitral annulus early diastolic velocity (LVEm) <8 cm/s than in those with an LVEm ≥ 8 cm/s (*p* < 0.01) (**B**).

However, there was no significant difference in LVEF between the dogs with SS/SS and control dogs (*p* > 0.05), 3 dogs with low LVEF (<50%) survived and all dead dogs had normal-supranormal LVEF (55%–86%) (Table 4, Figure 1A). Marked decreases in LVEDVI (decreased at admission), LVESVI (decreased at admission), and LVCI (decreased at admission, 6th and 24th hours) were observed in septic dogs compared to the control dogs. (*p* < 0.05) (Table 4). The experimental dogs’ LVEDVI, LVESVI, and LVCI reached the mean value with ongoing fluid therapy (Table 4). E and E/A in the dogs with SS/SS were decreased at admission when compared with the control dogs (*p* < 0.05) (Table 4). LVEm were decreased at admission, 6th, and 24th hours in the dogs with SS/SS when compared to control dogs (*p* < 0.05) (Table 4). 4 dogs with decreased LVEm died in the first 6 h of the treatment. Variables of diastolic dysfunction showed impaired relaxation (Grade I) in dogs with sepsis and the other types of diastolic dysfunction were not established. 10 of the 16 patients with LVDD on admission continued to exhibit signs of LVDD after 6 h of the treatment, while 2 of the patients still had LVDD after 24 h of treatment. Four dogs with LVDD died between the 2nd and 4th days of treatment. There was no statistical difference in EPSS, LVSm, LVAm, E/Em ratio, and A wave velocity between the experimental dogs and the control dogs during the study (*p* > 0.05) (Table 4). In the dogs with SS/SS, 18 dogs (78%) had at least one type of myocardial dysfunction. The LVSD and LVDD were present in 3 (13%) and 16 (70%) patients, respectively, and both types of dysfunction were present in 2 (9%) patients. Two dogs with SS/SS had neither LVSD nor LVDD.

### 3.7. Response to Treatment

Fifteen dogs with SS/SS recovered and were discharged from ICU. These dogs had a normal systolic and diastolic function on their final echocardiogram. 8 patients died (4 during the first 6 h, 4 between the 2nd and 4th days after the hospitalization), resulting in an overall mortality rate of 35%. The average period of survival was 1.3 ± 1.4 days for the non-survivor dogs (Figure 1A). None of the dogs with LVSD died, while 8 dogs with LVDD did not survive.

Two of the 3 patients with LVSD responded to the treatment during the first 6 h of the treatment and 1 patient still had LVSD after 24 h of treatment. Four dogs with LVDD died in the first 6 h of the treatment. LVDD was ongoing in 10 of the 16 dogs at the 6th and in 2 dogs at 24th hours of the treatment. The 4 dogs with LVDD died between the 2 and 4 days of the treatment. The LVEDVI, LVESVI, E, and E/A significantly increased in dogs with SS/SS within the first 6 h of initiating treatment (*p* < 0.05) (Table 4) and remained stable (at 6th and 24th hours of the treatment and on the D of D). LVCI significantly increased in dogs with SS/SS on the D of D (*p* < 0.05) (*p* < 0.05) (Table 4). LVEm remained low within the first 24 h of initiating treatment and reached the mean value of control dogs on the D of D (*p* < 0.05) (Table 4).

The HR significantly decreased in dogs with SS/SS within the first 6 h of initiating treatment (*p* < 0.05). CRT and PPQ normalized in dogs with SS/SS within the first 6 h of initiating treatment (*p* < 0.05). SpO2 in dogs with SS/SS was significantly decreased when compared with the value in the control group during the study (*p* < 0.05) (Table 1).

### 3.8. Survival Analysis

Kaplan–Meier’s analysis showed that the average time of the survivor was 108 ± 15 h. The cumulative survival probability was 73% ± 9%, 65% ± 9%, and 60% ± 10% at the 6th, 24th, and 48th hours of the study, respectively (Figure 1A). Kaplan–Meier analysis, along with the log-rank test, showed that the survival period was significantly shorter in patients with an LVEm < 8 cm/s when compared to those with an LVEm ≥ 8 cm/s (*p* < 0.01) (Figure 1B).

## 4. Discussion

Sepsis frequently affects the heart. Approximately 50% of the patients suffering from sepsis exhibit signs of SiMD in both humans and dogs [17,37]. Reversible SiMD has been recognized in 20% to 60% of humans with sepsis [38,39,40]. In our study, 78% of dogs (18/23) with SS/SS exhibited signs of myocardial dysfunction. 3 dogs (13%) with LVSD and 8 dogs (35%) with LVDD had reversible SiMD. The mortality rate in humans with sepsis who develop SiMD is higher than those without evidence of SiMD [41]. Eight (35%) dogs with LVDD died in our study.

The results of EExs (LVSD and LVDD), and SpO2, combined with the presence of increased CRT, HR, weak PPQ, and dehydration, hyperthermia/hypothermia, leukopenia, hemoconcentration, decreased BE, and increased ALT activities showed that circulatory dysfunction and impaired metabolism predominated in dogs with SS/SS in this study. These findings were consistent with those observed in dogs with SS/SS [17] and are an indicator of maldistribution of venous blood (relative hypovolemia) [42,43], impaired cellular metabolism [44,45], and SiMD [4,46].

The involvement of cardiac dysfunction in patients with SS/SS varies according to the timing and severity of the sepsis [47]. There is a consensus that LVEF is the most often used index for evaluating LVSD [23,28]. However, its association with the clinical outcome has given conflicting results [13,48]. Nelson and Thompson [17] reported that 75% of dogs with an LVEF of less than 46% died or were euthanized within 15 days of admission. However, it was found that reversible LVSD was associated with higher survival rates compared to those who had normal-supranormal LVEF in humans [15,49,50]. Many hypotheses have been suggested to explain why survivors exhibited more-marked myocardial depression [38,51,52]. To explain this situation, Levy et al. [53] have proposed that myocardial hibernation develops in sepsis. Myocardial hibernation is an adaptive mechanism to preserve cardiac myocytes by downregulation of oxygen consumption and energy requirements. By this action, cell-death pathway activation may decrease, and the future full recovery can start.

In our study, the 3 dogs with LVEF < 50% survived. All the non-survivor dogs had normal-supranormal LVEF. Jones et al. [54] stated that an echo derived LVEF > 55% was indicative of sepsis shock during the early phase of the disease. This could be explained by increased cardiac contractility due to adrenergic stimulation. However, despite this high LVEF, SV at this time point is low due to insufficient cardiac preload because of high vascular permeability and low vascular tone. The compensatory rise in HR is often insufficient to maintain adequate LVCO during this early phase of sepsis, as demonstrated by high lactate levels and a low central venous oxygen saturation [55]. Parallel to this, we determined low LVCI, high HR, and low SpO2 in dogs with SS/SS. Therefore, we think that low LVEF is an indication of a normal vascular tone. Two of the 3 patients with LVSD responded to the treatment during the first 6 h of the treatment and 1 patient with LVSD still had dysfunction at 24 h of the treatment. This finding shows that LVSD was reversible in septic dogs. The cTnI was not significantly different between control and experimental dogs in our study. This result can also explain that functional deterioration of LV, rather than structural damage, might be develop in septic dogs. Further studies are needed to confirm it.

Interestingly, we determined that 20 dogs with SS/SS had normal-supranormal LVEF. Dehydration rate was moderate (61%) and severe (39%) at admission in dogs with SS/SS. Hypotension was evident in 10 of the dogs with SS/SS at admission. Normal-supranormal LVEF can be explained by the balance between factors decreasing LVEF such as low preload and intrinsic alteration of contractility and parameters increasing LVEF such as decreased afterload, low blood pressure, and adrenergic stimulation. None of the dogs with LVSD died. For this reason, we assumed that low LVEF may be an indication of normal vascular tone and a good prognostic index. Therefore, normal-supranormal LVEF was not a useful index of LVSD in this case series, as there was no difference in LVEF between the control dogs and dogs with SS/SS. Thus, normal-supranormal LVEF may be observed in dogs with SS/SS, in whom arterial tone is usually decreased.

Recently, 2-dimensional speckle tracking echocardiographic (2D-STE) variables used to assess myocardial function in dogs with CPVI. Findings of impaired strain (St) and strain rate (SR) values in dogs with CPVI indicate the presence of systolic myocardial dysfunction in infected animals. This dysfunction may have been caused by direct viral action and/or the effects of SIRS on the myocardium [3]. In a study, Corda et al. [56] attempted to compare 2D-STE with 2D and M-mode echocardiography in the evaluation of systolic function in dogs with SIRS. They found that mild to moderate stages of SIRS in dogs were associated with LV systolic impairment identified by 2D-STE, but not detected by 2D- and M-mode-derived EF and FS. Both studies concluded that the evaluation of LV ventricular function by conventional echocardiographic indexes (EF and FS) is uncertain because these variables influenced by preload and CPVI patients are frequently dehydrated [22]. In addition, some technical limitations such as measure LV radial contraction without considering longitudinal and torsional deformation in FS and geometric assumption in M-mode derived EF prevent them from detecting mild decreases in systolic function [57]. Consequently, LVEF may be considered more as a “marker” of reduced vascular tone than of intrinsic LV contractility in dogs with SS/SS as demonstrated in critically ill humans [58,59].

When we evaluated the other indices for systolic function such as LVCI, EPSS and LVSm, there was no difference in EPSS and LVSm during the study, and LVCI was significantly low (at admission, 6th, and 24th hours) in the dogs with SS/SS compared to the control dogs. EPSS, similar to EF, may be considered a more relevant “marker” of reduced vascular tone than intrinsic LV contractility in dogs with SS/SS. This can change due to dehydration, hypotension, decreased LV afterload, and decreased LV preload. Decreased LVCI could be the result of decreased preload and decreased afterload as indicated by marked decreases in LVEDVI and LVESVI despite increased HR. The LVCI, LVEDVI, and LVESVI significantly increased in dogs with SS/SS within the first 6 h of initiating treatment and reached the mean value for control dogs with ongoing fluid therapy. The decreased LVEDVI and LVESVI showed that an absolute or relative loss of central blood volume (dehydration) on admission was an important cardiovascular derangement in the dogs with SS/SS [5]. Expansion of the extracellular fluid volume by the IV sodium-containing fluids appeared adequate in this study to address the preload restoration within the first 6 h of treatment. This observation suggested that periodic EExs of LVEDVI and LVCI would be clinically helpful in optimizing the rate of IV fluid administration in dogs with SS/SS.

With PW-TDI, the mitral annulus LVEm can be used to accurately assess LV relaxation [29,60]. Several studies have demonstrated that LVEm and variables obtained from TDI does not change significantly in response to different loading conditions [61,62,63,64,65], age and HR [66].

It has been reported that the lateral LVEm <10 and septal LVEm <8 cm/s highly suggestive of LVDD and elevated LA pressures (LAP) [67,68]. In humans, the studies showed that both LVSD and LVDD have developed in patients with SS/SS [16,69,70]. Landesberg et al. [8] informed that 9.1% of patients with SS/SS had isolated LVSD and 14.1% had combined LVSD and LVDD. However, the incidence of isolated LVDD was higher (38%). In the other studies, alarmingly high prevalence rates at 60–84% of LVDD with increased mortality have also been found in septic patients [8,12,71]. Ince et al. [4] found that LVEm, an index of LVDD, had the best sensitivity and specificity to differentiate survivor and non-survivor in septic dogs, with values of 100% (95% CI: 55.2–100) and 100% (95% CI: 78.9–100), respectively, at an optimum cut-off point of ≤6.50. Results of the present study showed that LVSD and LVDD were present in 13% and 70% of septic dogs, respectively and it is indicated that LVDD is a common phenomenon in dogs with SS/SS.

In humans, LVEm has prognostic importance in cardiac diseases [72]. Sturgess et al. [73] proposed that LVDD was an independent predictor of mortality, better than cardiac biomarkers. Landesberg et al. [8] investigated 262 patients with SS/SS using echocardiography and reported a 30% mortality rate within 30 days. LVDD may impair LV dilatation and prevent SV enhancement in response to fluid load. LVDD may also aggravate lung congestion. Developed non-cardiogenic pulmonary edema may lead to pulmonary hypertension and RV dysfunction. In our study, lower LVEDVI and LVCIs were seen in dogs with SS/SS and LVEm was strongly correlated with an adverse outcome. The survival period was significantly shorter in patients with an LVEm < 8 cm/s than in those with an LVEm ≥ 8 cm/s. Diastolic dysfunction is also characterized by increased LVFP (increased E, E/Em, and E/A). However, E and E/A decreased at admission, and E/Em in the dogs with SS/SS remained unchanged during treatment in our study. This could be explained by the development of distributive shock. Distributive shock occurs with the release of inflammatory mediators such as in sepsis or SIRS. Septic shock is subgroups of distributive shock [4,74]. It is commonly a complex process in which different mechanisms such as hypovolemia, vasoplegia, and septic cardiomyopathy may develop. The hyperkinetic LV can emerge in distributive shock. The decrease in LV afterload may mask LV dysfunction which may become obvious only after correction of hypotension [75]. Besides, hypovolemia is often consequently associated with a decrease in stressed volume related to venous dilation. In our study, the hyperkinetic LV (LVEF: 55–86%) combined with hypovolemia (decreased LVEDVI) was determined. This led to conflicting understand in the evaluation of LVFP. Hypovolemic shock develops secondary due to the lack of effective circulating blood volume [76]. Therefore, a significant decrease in LVEm and low E, E/A, and normal E/Em can be explained by the development distributive shock characterized with both septic cardiomyopathy and hypovolemic shock that present together in our study. A previous study in horses with SIRS demonstrated a mixed pattern combining impaired relaxation with the restrictive ventricular filling was suspected based on a higher E/Em ratio in the non-surviving horses [77]. However, our findings could not establish a significant difference in E/Em ratio in dogs with SS/SS, it seems that the absence of preload deficiency (normal EDVI) in horses with SIRS in contrast to dogs with sepsis that exhibited low EDVI may lead to the markedly increase in LVFP and E/Em in non-survivor horses. Thus, we suggest that the routine use of echocardiography is key to determine distributive shock in which both cardiac dysfunction and hypovolemia developed together. The conflicting effects of hemodynamic interventions must be considered.

Diastole is a complex mechanism during which various interrelated events lead to the ventricular filling before ejection. Mahjoub et al. [78] have performed research work to evaluate the improvement of LV relaxation as assessed by PW-TDI in fluid-responsive in septic shock. They found that LVEm maximal velocity increased with adequate volume expansion, suggesting an improvement of LV relaxation with the correction of hypovolemia in patients with septic shock. In our study, LVEm velocity increased by about 50 to 80 (60%) after volume expansion in dogs with SS/SS, corresponding to the enhancement of relaxation. Another explanation for augmentation of LV relaxation could be due to a phenomenon which is called the frequency-dependent acceleration of relaxation because of a decrease in HR [79]. A decrease in HR rather than an increase in HR would enhance relaxation [80]. Tachycardia, which is common in septic shock and is a known predictor of poor prognosis, promotes cardiac dysfunction by increasing oxygen requirements and diminishing diastolic cardiac filling and coronary perfusion [81].

In our study, the mean HR was significantly elevated in septic dogs at admission, and 6th hours when compared with the value in the control group, and the D of D. Previous studies have examined the effect of HR on survival. It is well established that HR plays an important role in cardiac function, and it has been shown in dogs that tachycardia-induced myocardial failure occurs with pacing >180 bpm [82,83]. In the present study decrease in HR after initiation of fluid therapy may be due to the fact that baro- and chemo-receptor activities are still preserved. A reduction in tachycardia could improve outcomes for septic patients by lowering cardiac workload and improving diastolic coronary perfusion of the septic heart [84].

## 5. Conclusions

LVDD, rather than LVSD, was the most clinically important cardiovascular abnormality in dogs with SS/SS. 8 dogs with LVDD, which accounts 50% dogs with LVDD, did not survive. Therefore, LVDD may be a guard prognostic index. LVSD was present in 13% of dogs. None of the dogs with LVSD died. Low LVEF in the early stages of SS/SS may be an indication of normal vascular tone (no vasoplegia) and provide a good prognostic index. The use of echocardiography for the monitoring of LVSD and LVDD in dogs with SS/SS is a useful ICU tool.

## Figures and Tables

**Table 1:** HR (mean ± standard deviation), CRT, PPQ, blood pressures (mean ± standard deviation) and SpO_2_ (%) (mean ± standard deviation), and SBP and MAP (mean ± standard deviation) in control dogs (*n* = 20) and the dogs with SS/SS (*n* = 23), during the experiment (at admission, at 6th and 24th hours of the treatment and on the D of D).

Variable	Control Group	Experimental Group			
		Admission	6 h	24 h	D of D
HR (bpm)	123.90 ± 23.83 ^c^	185.26 ± 34.79 ^a^	166.93 ± 28.46 ^ab^	143.33 ± 27.26 ^bc^	132.85 ± 26.13 ^c^
SpO_2_ (%)	86.90 ± 4.29 ^a^	47.85 ± 18.74 ^c^	63.64 ± 11.87 ^b^	58.01 ± 16.47 ^bc^	87.50 ± 14.98 ^bc^
SBP (mm Hg)	125.80 ± 17.02	107.86 ± 32.76	121.68 ± 21.36	122.86 ± 13.66	116.46 ± 12.19
MAP (mm Hg)	92.85 ± 16.92	78.34 ± 24.67	89.12 ± 17.02	90 ± 10	89.07 ± 7.96
CRT (s)	2 ^b^	3 ^a^	2 ^b^	1 ^b^	1 ^b^
PPQ	2 ^a^	1 ^b^	2 ^ab^	2 ^a^	2 ^a^

The same superscript letter on the same line indicated a non-significant difference between those group sharing the same latter. Different letter on the same line indicated significant difference (*p* < 0.05) between the groups. PPQ: Normokinetic (2); Hypokinetic (1); CRT: <2 s (1); 3–4 s (2); 4–5 s (3), HR: heart rate, CRT: capillary refill time, PPQ: peripheral pulse quality, SpO_2_: pulse oximetry SBP: systolic blood pressure, MBP: mean blood pressure.

**Table 2:** Hemogram parameters (mean ± standard deviation and median (min-max)) in control dogs (*n* = 20) and the dogs with SS/SS (*n* = 23), during the experiment (at admission, at 6th and 24th hours of the treatment and on the D of D).

Variable	Control Group	Experimental Group			
		Admission	6 h	24 h	D of D
WBC (cells/mL)	14.57 (7.75–20.34) ^ab^	4.55 (0.86–25.49) ^bc^	3.97 (0.98–21.87) ^c^	7.76 (1.41–21.60) ^bc^	17.74 (4.23–53.88) ^a^
Lymphocyte (cells/mL)	3.84 (1.25–11.43) ^ab^	3.29 (0.72–14.47) ^ab^	1.72 (0.59–13.07) ^a^	2.28 (0.98–3.53) ^a^	6.77 (3.10–22.30) ^b^
Monocyte (cells/mL)	1.17 (0.26–3.67) ^a^	0.13 (0.03–5.29) ^b^	0.12 (0.03–2.46) ^b^	0.29 (0.03–5.23) ^b^	2.56 (0.35–10.43) ^a^
Granulocyte (cells/mL)	8.73 (2.89–24.25) ^b^	1.27 (0.07–9.05) ^a^	2.30 (0.27–8.26) ^a^	4.13 (0.14–13.42) ^ab^	7.91 (0.78–21.56) ^b^
RBC (×10^3^ cells/mL)	5.86 ± 0.97 ^b^	7.36 ± 1.18 ^a^	6.59 ± 1.24 ^ab^	6.36 ± 1.01 ^ab^	6.17 ± 0.84 ^b^
MCV (fl)	56.61 ± 6.45 ^b^	61.10 ± 3.91 ^a^	60.45 ± 2.75 ^ab^	60.35 ± 3.23 ^ab^	59.21 ± 2.76 ^ab^
HCT (vol%)	33.30 ± 7.46 ^b^	45.04 ± 7.91 ^a^	39.75 ± 7.53 ^ab^	38.32 ± 6.13 ^ab^	36.42 ± 4.68 ^b^
MCHC (g/dL)	35.49 ± 16.44	29.56 ± 3.49	30.30 ± 4.40	32.35 ± 2.07	32.81 ± 2.36
Hgb (g/dL)	11.67 ± 1.12	13.61 ± 3.14	12.20 ± 3.26	12.42 ± 2.36	11.95 ± 1.61
Thrombocyte (cells/mL)	259.00 (84.00–804.00)	410.00 (57.00–1000)	318.50 (34.00–775.00)	361.00 (33.00–1002)	276.00 (49.00–905.00)

The same superscript letter on the same line indicated a non-significant difference between those group sharing the same latter. Different letter on the same line indicated significant difference (*p* < 0.05) between the groups. WBC: white blood cell count, RBC: red blood cell count, MCV: mean corpuscular volume, HCT: hematocrit, MCHC: mean corpuscular hemoglobin concentration, Hgb: hemoglobin.

**Table 3:** Acid-base balance and biochemical analysis parameters (mean ± standard deviation and median (min-max)) in control dogs (*n* = 20) and the dogs with SS/SS (*n* = 23), during the experiment (at admission, at 6th and 24th hours of the treatment and on the D of D).

Variable	Control Group	Experimental Group			
		Admission	6 h	24 h	D of D
pH	7.37 ± 0.03	7.33 ± 0.09	7.37 ± 0.07	7.39 ± 0.04	7.38 ± 0.04
pCO_2_ (mm Hg)	36.44 ± 3.66 ^ab^	41.80 ± 8.19 ^a^	34.42 ± 5.58 ^b^	37.77 ± 4.16 ^ab^	34.85 ± 5.24 ^b^
pO_2_ (mm Hg)	36.97 ± 5.94 ^a^	28.95 ± 6.89 ^b^	34.71 ± 4.76 ^ab^	31.72 ± 7.79 ^ab^	31.60 ± 8.38 ^b^
HCO_3_ (mmol/L)	22.74 ± 2.01	22.12 ± 5.85	19.98 ± 2.64	23.02 ± 2.84	21.14 ± 4.23
BE (mmol/L)	−2.35 (−4.20–3.50) ^a^	−6.00 (−14.90–12.80) ^b^	−4.40 (−12.30–1.00) ^b^	−2.00 (−6.30–5.20) ^ab^	−3.80 (−11.10–4.50) ^ab^
Na (mmol/L)	146.30 ± 6.22	141.69 ± 3.43	143.92 ± 4.87	144.06 ± 5.56	144.07 ± 6.90
K (mmol/L)	3.67 ± 0.49 ^a^	3.56 ± 0.52 ^ab^	3.04 ± 0.59 ^b^	3.18 ± 0.59 ^b^	3.57 ± 0.53 ^ab^
Lactate (mmol/L)	1.30 (0.60–2.00) ^ab^	1.90 (0.70–7.40) ^a^	1.05 (0.50–7.10) ^ab^	0.90 (0.60–3.00) ^b^	1.10 (0.70–2.80) ^ab^
Glucose (mg/dL)	93.80 ± 13.69	100 ± 32.96	96.35 ± 33.77	100.33 ± 24.82	85.38 ± 15.31
BUN (mg/dL)	10.50 (4.00–18.00)	11.00 (5.00–57.00)	9.00 (6.00–61.00)	14.00 (5.00–33.00)	13.00 (8.00–34.00)
Creatinine (mg/dL)	0.50 (0.30–1.10)	0.50 (0.30–2.60)	0.50 (0.40–2.80)	0.50 (0.30–4.00)	0.60 (0.40–1.00)
ALT (U/L)	27.00 (6.00–80.00) ^a^	40.00 (3.00–203.00) ^a^	22.00 (4.00–103.00) ^ab^	12.50 (7.00–144.00) ^b^	9.00 (4.00–52.00) ^b^
ALP (U/L)	213.00 (2.60–634.00)	272.00 (94.00–585.00)	274.00 (94.00–634.00)	288.00 (3.20–751.00)	246.50 (75.00–759.00)
Albumin (g/dL)	2.62 ± 0.45	2.53 ± 0.34	2.41 ± 0.21	2.35 ± 0.34	2.68 ± 0.51
Protein (g/dL)	5.74 ± 0.98	5.44 ± 0.71	5.18 ± 0.58	5.12 ± 0.77	5.35 ± 0.91
cTnI (pg/mL)	5.60 (2.40–29.30)	12.90 (1.80–85.10)	12.60 (1.70–88.40)	9.70 (1.90–49.60)	9.00 (1.10–110.90)

The same superscript letter on the same line indicated a non-significant difference between those group sharing the same latter. Different letter on the same line indicated significant difference (*p* < 0.05) between the groups. pH: blood pH, pCO_2_: blood partial carbon dioxide pressure, pO_2_: blood partial oxygen pressure, HCO_3_: bicarbonate, BE: base excess, Na: sodium, K: potassium, ALT: alanine amino transferase, ALP: alkaline amino transferase, cTnI: cardiac troponin I.

**Table 4:** Echocardiographic parameters (mean ± standard deviation) and cTnI (median (min-max)) in control dogs (*n* = 20) and in the dogs with SS/SS (*n* = 23) during the experiment (at admission, at 6th and 24th hours of the treatment and on the D of D).

Variable	Control Group	Experimental Group			
		Admission	6 h	24 h	D of D
EPSS (cm)	0.30 ± 0.08	0.23 ± 0.10	0.28 ± 0.12	0.30 ± 0.16	0.26 ± 0.07
LVEF (%)	63.34 ± 4.33	67.56 ± 13.42	63.12 ± 10.93	64.89 ± 10.79	69 ± 6.92
LVEDVI (mL/m^2^)	64.31 ± 17.76 ^a^	28.98 ± 11.34 ^b^	51.76 ± 12.29 ^a^	52.55 ± 13.27 ^a^	60.61 ± 15.02 ^a^
LVESVI (mL/m^2^)	22.07 ± 5.28 ^a^	12.05 ± 7.84 ^b^	18.93 ± 5.86 ^a^	18.62 ± 7.84 ^ab^	18.54 ± 7.20 ^ab^
LVCI (mL/min/m^2^)	5655 ± 1054 ^a^	2891 ± 985 ^d^	4010 ± 838 ^c^	4487 ± 1710 b ^c^	5217 ± 1216 ^ab^
LVSm (cm/s)	8.60 ± 1.41	9.86 ± 2.71	8.06 ± 2.66	8.59 ± 2.18	9.37 ± 2.63
LVEm (cm/s)	10.84 ± 1.39 ^a^	6.64 ± 1.76 ^b^	7.71 ± 3.16 ^b^	7.42 ± 2.61 ^b^	10.08 ± 2.29 ^a^
LVAm (cm/s)	6.43 ± 1.40	5.67 ± 1.68	6.70 ± 2.80	6.65 ± 1.71	7.01 ± 1.56
E/Em ratio	7.21 ± 0.89	6.96 ± 1.98	7.03 ± 0.84	7.92 ± 1.80	8.26 ± 1.72
E (cm/s)	78.39 ± 12.92 ^a^	50.53 ± 15.05 ^b^	71.52 ± 13.14 ^a^	74.68 ± 16.34 ^a^	81.19 ± 14.20 ^a^
A (cm/s)	53.06 ± 8.37	53.21 ± 21.00	55.03 ± 19.84	52.51 ± 12.32	52.73 ± 9.39
E/A ratio	1.46 ± 0.17 ^a^	1.04 ± 0.39 ^b^	1.27 ± 0.40 ^ab^	1.35 ± 0.38 ^ab^	1.55 ± 0.23 ^a^
EDT (s)	0.05 (0.04–0.07)	0.06 (0.04–0.11)	0.06 (0.04–0.11)	0.06 (0.03–0.09)	0.06 (0.03–0.08)

The same superscript letter on the same line indicated a non-significant difference between those group sharing the same latter. Different letter on the same line indicated significant difference (*p* < 0.05) between the groups. EPSS: E-point to septal separation, LVEDVI: left ventricle end-diastolic volume index, LVESVI: left ventricle end-systolic volume index, LVEF: left ventricle ejection fraction, LVCI: left ventricle cardiac index, LVSm: Left ventricle PW-TDI septal mitral annulus peak systolic velocity, E: PW-Doppler mitral inflow early diastolic peak velocity, A: PW-Doppler mitral inflow late diastolic peak velocity, E/A: E/A ratio, LVEm: left ventricle PW-TDI septal mitral annulus early diastolic velocity, Am: PW-TDI septal mitral annulus late diastolic velocity, E/Em: E/Em ratio, EDT: E-wave deceleration time.

## Data Availability

The data presented in this study are available on request from the corresponding authors.
